# Mental health and coping among graduate students during the COVID-19 pandemic: a gender-based analysis

**DOI:** 10.3389/fpsyt.2025.1532987

**Published:** 2025-07-03

**Authors:** Keira C. M. Aubin, Tara A. Thachet, Isabella Hotston, Ashley M. E. Thompson, Kim G. C. Hellemans

**Affiliations:** ^1^ Cumming School of Medicine, Department of Psychiatry, University of Calgary, Calgary, AB, Canada; ^2^ Department of Neuroscience, Carleton University, Ottawa, ON, Canada

**Keywords:** graduate students, COVID-19, coping, mental health, gender, stress, depression, anxiety

## Abstract

**Introduction:**

The COVID-19 pandemic has profoundly impacted the mental health of young adults in Canada, with research showing high rates of depression and anxiety symptomatology. Graduate students, who already experience elevated mental health challenges, represent a particularly vulnerable population—yet research examining their experiences during the pandemic remains limited. This study aims to investigate mental health and well-being outcomes, negative impacts, coping strategies, and gender differences among Canadian graduate students during the COVID-19 pandemic.

**Methods:**

A cross-sectional survey was conducted among Canadian graduate students (N = 261) to assess mental health symptoms, well-being, negative impacts, coping strategies, and gender differences during the COVID-19 pandemic using a series self-report of questionnaires (e.g. BDI, BAI, DASS-S). Data were analyzed using descriptive statistics, chi-square tests and t-tests.

**Results:**

Findings revealed significant mental health challenges among Canadian graduate students during the pandemic, with high rates of depression, anxiety, and stress reported. Female students reported worse mental health outcomes and experienced greater negative impacts compared to males. Coping strategies predominantly involved avoidant behaviors, such as watching TV and using social media, with gender differences in coping strategies.

**Discussion:**

Compared to pre-pandemic findings, graduate students in this pandemic sample reported elevated rates of mental health challenges. Women appeared to be disproportionately impacted, reflecting the heightened mental health burden they reported during this period. Avoidant coping strategies were most commonly used—aligning with the socially isolating conditions of the pandemic—with notable gender differences in types of strategies employed.

**Conclusion:**

The COVID-19 pandemic seems to have exacerbated the mental health crisis among Canadian graduate students, with higher rates of depression, anxiety, and stress reported compared to pre-pandemic findings. Female students face heightened challenges, emphasizing the need for gender-sensitive support strategies. Universities should prioritize mental health support and promote healthy coping mechanisms to address the impacts of the pandemic on graduate student well-being.

## 1 Introduction

### 1.1 Prevalence and risk factors in graduate student mental health

Over the last decade, growing attention has been drawn to the mental health of graduate students, with increasing concern surrounding high levels of psychological distress - particularly suicidal ideation (1–3). For example, Evans et al. (1) found that 41% of graduate students experienced moderate to severe anxiety, and 39% met the criteria for moderate to severe depression - compared to just 6% in the general population, suggesting possible enduring implications for graduate students’ mental well-being. Mental health challenges among graduate students are closely linked with reduced academic performance, impaired personal development, diminished productivity, and compromised quality of life (1, 3).

Data from the National College Health Assessment (NCHA) further shows concerning trends. In 2013, 91.7% of students reported feeling overwhelmed, 89.5% felt exhausted, 70.0% felt lonely, 60.4% experienced overwhelming anxiety, 38.6% felt so depressed it was difficult to function, and 10.0% had seriously considered suicide. By 2019, some of these numbers had escalated. Of the 13,000 graduate students surveyed, 64% rated their stress levels over the past year as more than average or tremendous (4). Specifically, 88.2% felt overwhelmed, 87.6% felt exhausted, 69.6% felt lonely, 68.9% felt overwhelming anxiety, 51.6% felt so depressed it was difficult to function, and 16.4% had seriously considered suicide. These alarming trends point not only to an immediate mental health crisis but also underscore significant potential long-term implications, emphasizing urgent need for improved support systems, effective interventions, and longitudinal research.

While stressors such as financial pressures, limited support, and insufficient time to establish careers are common among young adults aged 21-30 (5, 6), including both undergraduate (7, 8) and graduate students (9), graduate students also face a distinct set of challenges (10, 11). Researchers have identified them as particularly vulnerable to work-life imbalance, largely due to the difficulty of balancing intense academic demands with personal responsibilities, such as caregiving or maintaining relationships (10, 12). Other contributing factors to the high prevalence of mental health issues among graduate student include strained supervisor-student relationships, inadequate peer support, limited career prospects, a perceived lack of control, and the inherently competitive nature of graduate programs (3). These combined pressures may exacerbate their vulnerability to long-term mental health issues, and worsened quality of life.

### 1.2 COVID-19 disruptions and consequences for Canadian graduate students

While the COVID-19 pandemic caused global disruption at all levels of education, graduate students may have been uniquely impacted. In Canada, the COVID-19 pandemic led to abrupt and challenging adjustments for students and institutions. Following the government’s declaration of a national emergency in mid-March 2020, Canadian universities and colleges suspended all in-person classes and rapidly transitioned to remote learning platforms (13). As the pandemic persisted, some programs introduced hybrid formats starting in Fall 2020, particularly for courses with essential lab or hands-on components. However, most Canadian campuses did not resume in-person learning until Fall 2021 (14, 15), prolonging uncertainty for students.

Public health measures such as lockdowns, travel bans, and the closure of schools (13) forced a rapid shift to remote learning and the abrupt closure of campuses (16). For graduate students, this meant not only interrupted coursework but also restricted access to critical academic infrastructure, including research labs (14, 17). Although both undergraduate and graduate students experienced elevated rates of depression and anxiety during the pandemic (18) one study found that undergraduates reported greater overall mental health impacts (19). Still, graduate students face distinct burdens. Their advanced training often relies on in-person research, and the closure of labs and suspension of fieldwork left many unable to progress on dissertations or publications (17). Furthermore, the limited lab access and closure of campuses also caused financial instability for many graduate students, as most rely heavily on their positions as research and teacher’s assistants or grant funded projects (20, 21). Many expressed concerns over reduced funding opportunities, the inability to travel or complete field/lab work, and diminished career prospects (16). At Vanderbilt University, one-third of graduate students reported mental health declines tied to disrupted lab access, delayed degree timelines, and reduced mentorship (14). Moreover, while undergraduates could transition most coursework online, the nature of graduate programs, reliant on specialized equipment, field/lab sites, and close supervisor interactions, meant that remote formats were often inadequate substitutes (16). Graduate students who met the criteria for depression and anxiety also reported the most difficulty adjusting to online learning. In mid-2020, the Student Experience in the Research University (SERU) Consortium found that 32% of graduate and professional students screened positive for major depressive disorder - twice the rate from 2019 - and 39% screened positive for generalized anxiety disorder, a 1.5-fold increase from the previous year (18). While these rates are similar to those reported in 2018 (1), they remain alarmingly high, suggesting that the pandemic may have intensified, or at least sustained, the ongoing graduate student mental health crisis, with potential implications for retention.

At the same time, graduate students possess advanced applied skills, discipline-specific training, and autonomy compared to undergraduates. These attributes can enable them to navigate complex research environments, engage in independent learning, and manage self-direct work effectively (22). Given these competencies, one might expect graduate students to be better equipped to adapt to challenges posed by the COVID-19 pandemic, particularly in managing remote work or progressing through reading- or writing-intensive tasks. However, the ability to persist was far from uniform. Disparities in access to resources, support systems, and research infrastructure created uneven impacts across disciplines and individual circumstances. When considered alongside pre-existing challenges, and the documented rise in graduate student suicidal ideation between 2013 and 2019 (4, 23), it is unsurprising that the pandemic may have further intensified mental health struggles in this already vulnerable population (14, 16, 24).

Graduate students are the future of academia and highly skilled professions, yet high attrition rates are not uncommon (25). Between 2004 and 2010, graduate students enrolled in Great Plains IDEA-sponsored online master’s programs showed a dropout rate of 25% and a retention rate of 67.7%. Nationally, data from the Council of Graduate Schools show that 23% of STEM masters students withdrew within two years in 2015, while only 57% of doctoral candidates completed their programs within ten years - a 43% attrition rate (26, 27). These persistently high rates of attrition, alongside COVID-19 disruptions, financial strains, and restricted access to research infrastructure, highlight the growing risk of losing a critical pipeline of scholars poised to shape the future of knowledge, research, and academia.

### 1.3 Gender disparities in graduate student mental health and academic outcomes

While most graduate students are affected by mental health challenges, these issues tend to be more pronounced among women (1, 28, 29), despite women being more likely than men to pursue both undergraduate and graduate education (30). Evans et al. (1) identified clear gender-based disparities in mental health outcomes for graduate students: 34% of men reported experiencing moderate to severe anxiety, compared to 43% women. Similarly, 41% of women met the criteria for moderate to severe depression on the PHQ-9 scale, versus 35% of men. These differences are likely driven, at least in part, by persistent gender bias in academia, particularly in STEM fields, where women frequently face barriers in securing research funding, mentorship, and career advancement opportunities (31–33). For instance, studies suggest women researchers often receive smaller research grants and fewer funding awards compared to male counterparts, potentially limiting their professional growth and exacerbating stress levels (34). Additionally, women in STEM and graduate programs are more likely to assume greater family and caregiving responsibilities, which further compound their mental health and work-life balance challenges, ultimately impacting their academic productivity and career progression (35). Although women now represent a larger portion of students in higher education, gender disparities remain more pronounced in higher academic positions. For example, in the NIH intramural research program, women held 37% of tenure-track roles but accounted for only 21% of those granted tenure. Women of color were especially underrepresented, comprising just 5% of tenured faculty (36).

### 1.4 Transactional model of stress and coping: a framework for understanding graduate student well-being

This study is grounded in Lazarus and Folkman’s (37) transactional model of stress and coping, which provides a theoretical framework for understanding how individuals assess and respond to stress. According to this model, coping involves two key processes: (1) the appraisal of a stressor’s significance, and (2) the selection of coping strategies, broadly categorized as problem-focused or emotion-focused. While this framework has been widely applied to undergraduate populations (38, 39), there remains limited empirical research exploring how graduate students, who face distinct stressors, apply coping strategies, particularly in the context of large-scale disruptions such as the COVID-19 pandemic. The present study applies this model to investigate coping behaviours and mental health outcomes among graduate students, with attention to gender differences in coping style.

While women are more likely to seek help and engage in social coping strategies, men may be less inclined to seek support and more prone to intense emotional episodes, such as breakdowns (29, 40–42). Supporting this, Ickes et al. (43) found that female graduate students relied more heavily on social support compared to male graduate students and undergraduates. These gendered patterns in mental health symptoms and coping behaviours highlight the need for further research that may support more nuanced research-backed support structures in graduate education. Despite the substantial evidence outlined above, mental health and coping among graduate students, particularly as a function of gender, remains significantly under-researched within a Canadian context. This study seeks to address this critical gap by examining graduate student mental health and coping strategies during the COVID-19 pandemic, with a focus on gender differences.

### 1.5 Study aims and hypotheses

The primary aim of this exploratory study is to investigate how graduate student mental health and well-being may have been affected by the COVID-19 pandemic. The primary hypothesis for this study is that Canadian graduate students will report mental health challenges at rates exceeding those documented in pre-COVID data, and that women will be disproportionately affected compared to men. In particular, social connectedness will emerge as one of the more severely impacted domains of well-being. With respect to coping strategies, it is hypothesized that avoidant coping mechanisms will be most employed, specifically watching television, using social media, and playing video games - behaviours that align with the isolating conditions of the pandemic. Furthermore, gendered patterns in coping mechanisms are predicted: women will more often report using social media, while men will more often report playing video games to cope. By exploring these dimensions, this study aims to contribute critical insights into the gendered nuances of graduate student mental health and coping during one of the most globally disruptive periods in recent history.

## 2 Methods

### 2.1 Procedure

To recruit participants from all disciplines, email invitations were sent to the graduate student societies and administrators at Canadian universities, posters were shared on the social media accounts of members of the research team, and snowballing methods were used. The survey was administered from April-May 2021, and all participants were entered into a draw for a chance to win 1 of 40 Amazon gift cards valued at $50 each. Once consent was obtained, participants completed an online survey through Qualtrics (Qualtrics Provo, UT) which contained demographic questions (e.g., age, sex/gender, discipline of study) and a series of questionnaires and scales related to mental health, coping, and substance use. This study was cleared by the Carleton Research Ethics Board (REB # 115344).

### 2.2 Participant demographics

The participants in this study (N = 261; 65.9% female) were graduate students in any discipline who were enrolled at a Canadian University (M_age_ = 28.2 years, range = 20–59 years), and there were no exclusion criteria. The average number of years spent as a graduate student was 2.5 years (range = 0–10 years) and 32.2% of the participants (*n* = 84) had a diagnosed mental health disorder. Please see **Table 1** to view additional demographic information for the participants in this study.

**Table 1:** Demographic data of the study population.

Demographic categories	Frequency (%)
Gender (n=261)	
Female (n=172)	65.9
Male (n=79)	30.3
Non-binary (n=6)	2.3
Gender non-conforming (n=2)	0.8
Gender queer (n=1)	0.4
Other (n=1)	0.4
Ethnicity (n=261)	
White North-American (n=128)	49.0
White European (n=46)	17.6
Asian (South Asia, Arab/West Asian, East Asian, and Indian Caribbean, n=48)	18.4
Other/Mixed Ethnicity/Prefer not to answer (n=14)	5.3
Latin American/Hispanic (n=11)	4.2
Middle Eastern (n=7)	2.7
Black (Black African, Black North American, and Black Caribbean, n=5)	2.0
Indigenous (Aboriginal, Metis, First Nations, and Inuit, n=2)	0.76
Graduate student disciplines (n=261)	
Social Sciences/Humanities (n=93)	35.6
Life Sciences (n=84)	32.2
Engineering (n=58)	22.2
Public Affairs (n=18)	6.9
Prefer not to answer (n=5)	1.9
Business (n=3)	1.1
Current employment status (n=261)	
Part-time (n=106)	40.6
Unemployed (n=95)	36.4
Full-time (n=40)	15.3
Other (n=18)	6.9
Retired (n=1)	0.4
Prefer not to answer (n=1)	0.4

### 2.3 Measures

#### 2.3.1 Demographics

Participants provided information on their basic demographic details including sex/gender identity, age, ethnicity, year of study, and living situation. Additionally, they responded to inquiries concerning their overall mental and physical well-being.

#### 2.3.2 Anxiety, depression, and stress symptoms

The Depression, Anxiety, and Stress Scale (DASS; 44) is a 21-item scale that consists of 3 subscales that measure the emotional states of depression, anxiety, and stress over the past week. Each question in the scale has a range of 4 options that indicate low (0) to high (3) depressive, anxiety, and stress symptoms. In this thesis, the stress subscale of the DASS was analyzed (DASS-S). Total scores for the subscale were summed and total scores were placed in the following categories as per the DASS-S: 0-7 (normal stress), 8-9 (mild stress), 10-12 (moderate stress), 13-16 (severe stress), and 17+ (extremely severe). The DASS-S had high internal reliability (*α* = 0.88). Prior research with higher education populations has demonstrated that the DASS possesses excellent internal validity (45).

##### 2.3.2.1 Anxiety

The 21-item Beck Anxiety Inventory (BAI; 46) is comprised of 21 questions to assess anxiety symptomology over the past week. Scores range from low (0) to high (3) anxiety symptomology. The scores were summed, and higher scores reflected greater anxiety symptomology. Total scores were placed in the following categories as per the BAI: 0-7 (minimal anxiety), 8-15 (mild anxiety), 16-25 (moderate anxiety), and 26-63 (severe anxiety). The scale had high internal reliability (*α* = 0.93). The BAI scale has demonstrated strong internal consistency across studies involving similar populations (47, 48).

##### 2.3.2.2 Depression

The 21-item Beck Depression Inventory (BDI; 49) is comprised of 21 questions, each including a range of 4–5 options to assess depressive symptomology over the past week. Scores range from low (0) to high (3) depressive symptomology. The scores were summed and total scores were placed in the following categories as per the BDI: 0-13 (minimal depression), 14-19 (mild depression), 20-28 (moderate depression), and 29-63 (severe depression). The scale had high internal reliability (*α* = 0.91). The BDI has demonstrated strong internal consistency in previous studies examining comparable populations (50, 51).

##### 2.3.2.3 Stress

The 21-item University Stress Scale (USS; 52) was used to measure the level of stress experienced by university students. Each question in the scale had a range from 0 (not at all) to 3 (constantly). Total scores were summed higher scores reflected higher stress associated with university. Total scores were placed in the following categories as per the USS: 0-12 (minimal psychological distress) and 13+ (significant psychological distress). The scale had high internal reliability (α = 0.85). The USS has shown strong internal consistency in prior research involving university student populations (53).

#### 2.3.3 The COVID-19 Pandemic Questionnaire

The research team developed a COVID-19 Pandemic Questionnaire (adapted from 54) to measure how the pandemic affected the life, activities, thoughts, and mood of graduate students. Due to the urgent and exploratory nature of data collection during the early stages of the pandemic, no formal pilot testing or psychometric validation of the adapted COVID-19 Pandemic Questionnaire was conducted. As such, its use in this study is intended to provide descriptive insights rather than infer validated constructs. The questionnaire was divided into two sections. The first section assessed the negative impact of the pandemic on graduate student life over the past month (e.g., mental health, stress levels, academic performance, and social relationships). Participants rated each item on a 5-point Likert scale ranging from 1 (not at all) to 5 (an extreme amount). The second section assessed the frequency with which students engaged in various coping strategies to manage pandemic-related stress. This included 14 specific behaviours adapted from Prowse et al. (54), such as watching TV, exercising indoors, eating fast food/sweets, using social media, gaming, talking to friends/family, meditating, and engaging in creative hobbies. Participants rated each coping strategy on a 5-point Likert scale in response to the question, “To what extent have you been doing X to cope with the COVID-19 pandemic?” (1 = not at all; 5 = an extreme amount). All coping items were analyzed individually; no aggregate subscales or categories (e.g., avoidant vs. approach coping) were created. This approach allowed us to identify the most commonly used strategies and explore potential gender differences in specific behaviours.

### 2.4 Statistical analysis

All statistical analyses were performed with IBM SPSS Statistics for MacOS, Version 28 (IBM Corp., Armonk, NY., USA). Participants who completed the study in 1200 seconds or less were omitted from the study. Various validity checks were conducted to ensure the data was complete and reliable. Due to insufficient sample sizes for gender, the ten participants who did not identify as man or woman were excluded from all analyses. Responses from the COVID-19 pandemic questionnaire were grouped into ordinal categories: (1) not at all/a little, (2) a moderate amount, and (3) very much/an extreme amount. Chi-squared analyses were used to examine the relationship between gender and the COVID-19 pandemic questionnaire. Effect sizes were evaluated using Cramér’s V coefficients. For significant chi-squared results, Bonferroni corrections were applied to identify which cells significantly differed from one another. Independent samples *t*-tests were used to assess mean differences by gender in continuous mental health outcomes. Effect sizes were reported using Cohen’s *d*. A one-way multivariate analysis (MANOVA) was conducted to assess gender differences in different mental health outcomes. Prior to analysis, assumptions were assessed, including normality, linearity, multicollinearity, and homogeneity of variance. Bootstrapping procedures with 5000 resamples and bias-corrected and accelerated (BCa) 95% confidence intervals (CI) were employed to increase estimate robustness for all tests. Statistical significance was determined at *p < 0.05* (two-tailed).

An *a priori* power analysis was conducted using G*Power (55) to determine the minimum sample size required to detect a medium multivariate effect (*f^2^* = 0.068; based on observed *η²* = 0.064), with *α* = 0.05, *power* = 0.08, two groups, and three dependent variables. The analysis indicated that a minimum of 166 participants was required. The final sample of 251 participants exceeded this requirement.

## 3 Results

### 3.1 Mental health symptoms

The total percent of participants with anxiety symptomology was 57.9% (*n* = 151). For BAI (*n* = 261), 42.1% of participants had minimal anxiety (*n* = 110), 29.1% had mild anxiety (*n* = 76), 18.8% had moderate anxiety (*n* = 49), and 10.0% had severe anxiety (*n* = 26). There was a significant relationship between BAI symptom severity and gender, *χ^2^* (3, *n* = 251) = 17.82, *p <.001*, Cramér’s *V* = 0.27, 95% BCa CI [0.15, 0.40]). *Post hoc* analysis of standardized residuals indicated that in comparison to males, females were more likely to report *severe anxiety* and less likely to report *minimal anxiety.* Overall mean scores also indicated that females reported higher levels of anxiety, *t*(249) = -3.71, *p*<.001, 95% BCa CI [-7.95, -2.88], d = -0.50. compared to males. For BDI (*n* = 261), the total percent of participants with depressive symptomology was 70.4% (*n* = 184). 29.5% of participants had minimal depression (*n* = 77), 35.6% had mild depression (*n* = 93), 24.5% had moderate depression (*n* = 64), and 10.3% had severe depression (*n* = 27). While there were no gender differences between males and females for each category, overall mean scores were significantly different as females reported higher levels of depression, *t*(249) = -2.53, *p* = 0.01, 95% BCa CI [-5.77, -0.78], *d* = -0.34.

For DASS-S (*n* = 261), the total percent of participants with general stress symptomology was 49.8% (*n* = 130). 50.2% of participants had normal stress (*n* = 131), 24.5% had mild stress (*n* = 64), 16.5% had moderate stress (*n* = 43), 5.0% had severe stress (*n* = 13), and 3.8% had extremely severe stress (*n* = 10). There was a significant relationship between DASS-S symptom severity and gender, *χ^2^* (4, *n* = 251) = 10.68, *p* = 0.03, Cramér’s *V* = 0.21, 95% BCa CI [0.12, 0.36]). Specifically, *post hoc* analysis of standardized residuals indicated that in comparison to males, females were more likely to report extremely severe stress and were less likely to report *normal stress*. Overall mean scores also indicated that females reported higher levels of general stress, *t(249) = -3.75, p<.001*, 95% BCa CI [–7.19, -2.48], *d* = -0.51, compared to males. For USS (*n* = 261), 33.3% of participants had minimal psychological distress (*n* = 87) and 66.7% experienced significant psychological distress (*n* = 174). A significant relationship between USS symptom severity and gender was observed, *χ^2^* (1, *n* = 251) = 8.09, *p < 0.01*, Cramér’s *V* = 0.18, BCa 95% CI [0.05, 0.31]). *Post hoc* analysis of standardized residuals indicated that in comparison to males, females were more likely to report significant psychological distress. Similarly, overall mean scores also indicated that females reported higher levels of university stress, *t*(249) = -2.54*, p* = 0.01, BCa 95% CI [-5.65, -0.72], *d* = -0.35, compared to males.

A one-way multivariate analysis (MANOVA) was conducted to explore gender differences in stress (DASS_Stress), depression (BDI), and anxiety (BAI). The test revealed a significant effect of gender, Wilks’ λ = 0.94, *F*(3, 247) = 5.67, p > 0.001, η² = 0.06. Univariate ANOVAs indicated that females reported significantly higher stress scores than males, *F*(1, 249) = 6.38, p = 0.01, η² = 0.03. Females also reported higher levels of anxiety in comparison to males, *F*(1, 249) = 13.75, p < 0.001, η² = 0.05.

### 3.2 Stressors affecting graduate students during the COVID-19 pandemic

Data from the University Stress Scale (USS) revealed that the top 5 stressors constantly affecting graduate students were: academic/course work demands (39.5%), procrastination (36.4%), study/life balance (28.4%), mental health problems (21.8%), and work (16.5%); see **Table 2**. There was a significant relationship between academic/course work demands and gender, *χ^2^* (3, *n* = 251) = 8.94, *p* = 0.03, Cramér’s *V* = 0.19, 95% BCa CI [0.06, 0.37]. Specifically, *post hoc* analysis of standardized residuals indicated that in comparison to men, a greater proportion of women were more likely to report that they were affected by the stressor *constantly* and less likely to report that they were affected by the stressor sometimes. There was no significant relationship between procrastination and gender, *χ^2^* (3, *n* = 251) = 6.15, *p* = 0.11, Cramér’s *V* = 0.16, 95% BCa CI [0.03, 0.34]. There was a significant relationship between study/life balance and gender, *χ^2^* (3, *n* = 251) = 10.49, *p* = 0.02, Cramér’s *V* = 0.20, BCa 95% CI [0.07, 0.37]. Specifically, *post hoc* analysis of standardized residuals indicated that in comparison to men, a significantly greater proportion of women reported being *frequently* affected by the stressor, while men were more likely to report being sometimes affected. There was a significant relationship between mental health problems and gender, *χ^2^* (3, *n* = 251) = 11.80, *p* = 0.01, Cramér’s *V* = 0.22, 95% BCa CI [0.09, 0.38]. Specifically, *post hoc* analysis of standardized residuals indicated that in comparison to men, a greater proportion of women were more likely to report that they were constantly affected by the stressor and less likely to report that they were not at all affected by the stressor. There was no significant relationship between work and gender, *χ^2^* (3, *n* = 251) = 4.54, *p* = 0.21, Cramér’s *V* = 0.13, 95% BCa CI [0.02, 0.35].

**Table 2:** Frequency of the top 5 stressors for graduate students over the COVID-19 pandemic as well as gender differences from the USS.

Stressor	Not at all (%)	Sometimes (%)	Frequently (%)	Constantly (%)
Academic/course work demands* (n= 261)	5.0 (n=13)	24.9 (n=65)	30.7 (n=80)	39.5 (n=103)
Males (n=79)	5.1 (n=4)	36.7 (n=29)	27.8 (n=22)	30.4 (n=24)
Females (n=172)	5.2 (n=9)	19.8 (n=34)*	30.8 (n=53)	44.2 (n=76)*
Procrastination (n=261)	10.0 (n=26)	25.3 (n=66)	28.4 (n=74)	36.4 (n=95)
Males (n=79)	8.9 (n=7)	35.4 (n=28)	24.1 (n=19)	31.6 (n=25)
Females (n=172)	10.5 (n=18)	20.9 (n=36)	32.0 (n=55)	36.6 (n=63)
Study/life balance* (n=261)	15.7 (n=41)	26.1 (n=68)	29.9 (n=78)	28.4 (n=74)
Males (n=79)	21.5 (n=17)	35.4 (n=28)	20.3 (n=16)	22.8 (n=18)
Females (n=172)	13.4 (n=23)	22.1 (n=38)*	33.7 (n=58)*	30.8 (n=53)
Mental health problems* (n=261)	29.1 (n=76)	26.4 (n=69)	22.6 (n=59)	21.8 (n=57)
Males (n=79)	41.8 (n=33)	30.4 (n=24)	15.2 (n=12)	12.7 (n=10)
Females (n=172)	24.4 (n=75)*	26.2 (n=69)	25.0 (n=55)	24.4 (n=52)*
Work (n=261)	28 (n=73)	31.8 (n=83)	23.8 (n=62)	16.5 (n=43)
Males (n=79)	36.7 (n=29)	26.6 (n=21)	21.5 (n=17)	15.2 (n=12)
Females (n=172)	23.8 (n=41)	33.7 (n=58)	1.4 (n=42)	18.0 (n=31)

* *p*<0.05

### 3.3 Most common negative impacts of COVID-19 among graduate students by gender

**Figure 1:**
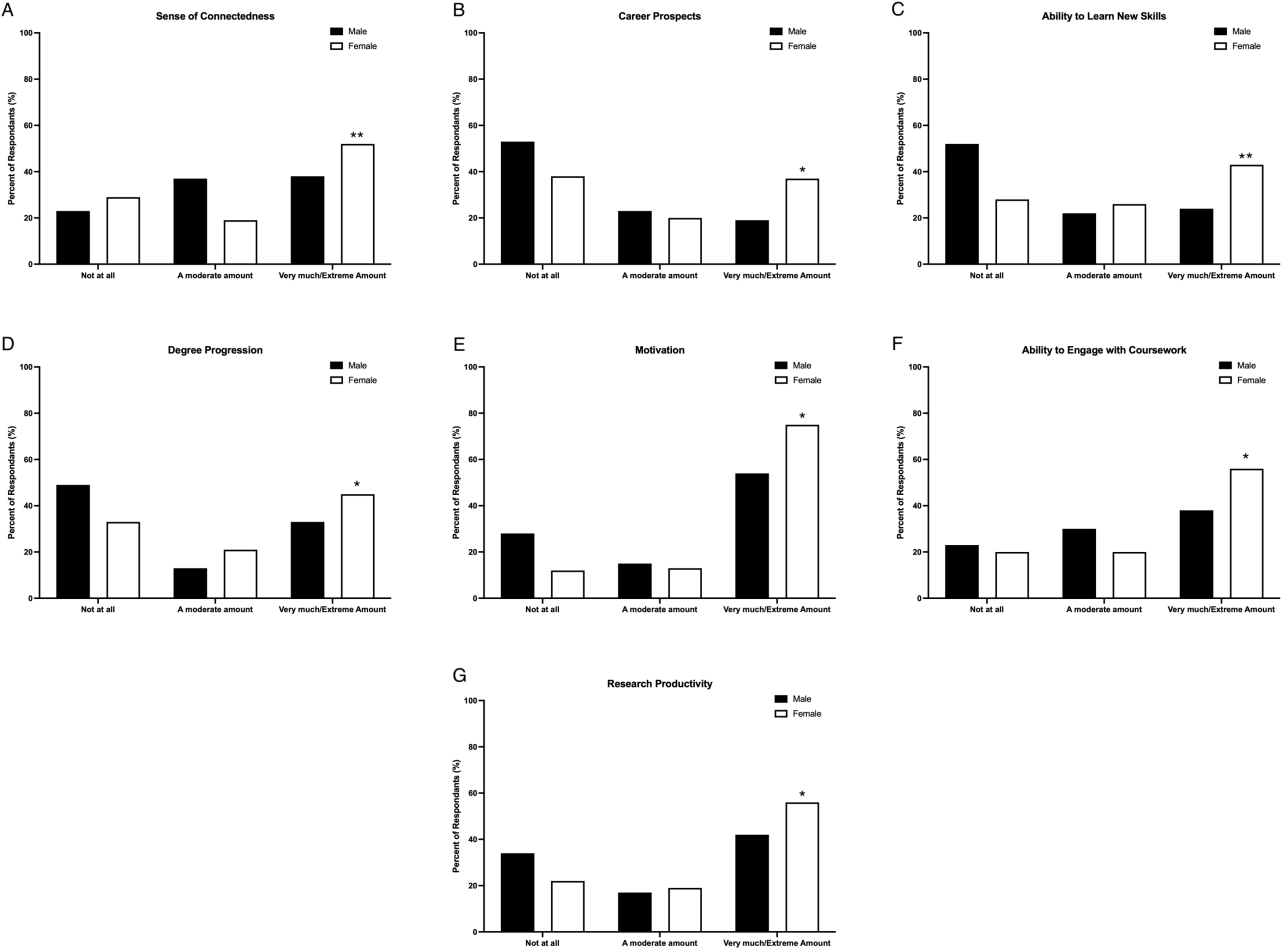
The top negative impacts of the COVID-19 pandemic on graduate students by gender included **(A)** sense of connectedness, **(B)** career prospects, **(C)** ability to learn new skills, **(D)** degree progression, **(E)** motivation, **(F)** ability to engage in course work, and **(G)** research productivity. *p < 0.05 relative to males within the same category, **p < 0.001 relative to males within the same category.

The top five negative impacts of the pandemic that affected graduate students were: stress levels (35.2%); mental health (34.1%); social relationships (31.8%); overall educational experience (31.0%); and sense of connectedness with family members/friends/loved ones (28.7%). Gender significantly influenced all outcome variables (See **Figures 1A–G**). Specifically, *post hoc* analysis of standardized residuals indicated a greater proportion of women were more likely to report the pandemic impacted the following outcomes very much/an extreme amount compared to men: their sense of connectedness with family members/friends/loved ones, *χ^2^* (5, *n* = 251) = 17.00, *p* = 0.01, Cramér’s *V* = 0.26, 95% BCa CI [0.12, 0.45]; career prospects, *χ^2^* (5, *n* = 251) = 12.86, *p* = 0.03, Cramér’s *V* = 0.23, 95% BCa CI [0.09, 0.42]; their ability to learn new skills/techniques, *χ^2^* (5, *n* = 251) = 15.85, *p* = 0.01, Cramér’s *V* = 0.25, 95% BCa CI [0.11, 0.46]; degree progress on, *χ^2^* (5, *n* = 251) = 13.96, *p* = 0.02, Cramér’s *V* = 0.24, 95% BCa CI [0.10, 0.44]; motivation, *χ^2^* (5, *n* = 251) = 16.02, *p* = 0.01, Cramér’s *V* = 0.25, 95% BCa CI [0.11, 0.44]. There was no significant relationship between gender and ability to engage in coursework, *χ^2^* (5, *n* = 251) = 10.89, *p* = 0.05, Cramér’s *V* = 0.21, 95% BCa CI [0.06, 0.44] and between gender and research productivity, *χ^2^* (5, *n* = 251) = 8.85, *p* = 0.11, Cramér’s *V* = 0.19, 95% BCa CI [0.06, 0.46].

### 3.4 Most common coping strategies used by graduate students to cope with the stressors of COVID-19 by gender

**Figure 2:**
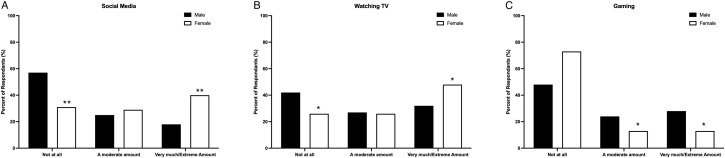
The top coping strategies used by graduate students to cope with the stressors of the COVID-19 pandemic by gender included **(A)** gaming, **(B)** social media, and **(C)** watching TV. *p < 0.05 relative to males within the same category, **p < 0.001 relative to males within the same category.

The top coping strategies endorsed by graduate students were: watching TV (32.2%); social media (25.7%); working (21.5%); connecting with friends and family members through videoconferencing methods (21.1%); exercising indoors (20.3%); eating fast foods/sweets (19.2%); and sleeping (17.2%). There was a significant relationship between social media and gender, *χ^2^* (4, *n* = 251) = 20.57, *p* < 0.001, Cramér’s *V* = 0.29, 95% BCa CI [0.16, 0.45], where a greater proportion of women were more likely to report that they used social media by very much/an extreme amount and less likely to report that they used social media not at all or a little (See **Figure 2A**). The relationship between watching TV and gender was also observed as significant, *χ^2^* (4, *n* = 251) = 10.70, *p* = 0.03, Cramér’s *V* = 0.21, 95% BCa CI [0.08, 0.40], where a greater proportion of women were more likely to report that they watched TV very much/an extreme amount and less likely to report that they did not watch TV to cope not at all/a little (See **Figure 2B**). Lastly, gender significantly influenced the impact of employing gaming as a coping strategy, *χ^2^* (4, n = 251) = 18.20, p < 0.001, Cramér’s V = 0.27, 95% BCa CI [0.12, 0.46]. Specifically, men were more likely to report that they engaged in gaming a moderate amount or by very much/an extreme amount as a coping strategy compared to women (See **Figure 2C**).

## 4 Discussion

The COVID-19 pandemic significantly impacted the mental health of young adults in Canada. During the Spring of 2021, approximately 1 in 4 young adults over the age of 18 screened positive for symptoms of depression, anxiety, or PTSD. In addition, 80% of adults aged 25–44 reported at least one negative impact from the pandemic (56). Graduate students often fall within this age demographic, and their mental health has been recognized as a growing crisis for years prior to the pandemic, highlighting a critical need to understand and address both immediate and longer-term mental health outcomes (1). Yet, there remains limited research on how the COVID-19 pandemic specifically affected graduate students’ mental health and well-being. This study aimed to expand the existing literature to explore mental health outcomes among Canadian graduate students during the pandemic, including negative impacts, coping strategies, and the potential influence of gender on these outcomes.

### 4.1 Mental health outcomes

Self-reported data from this study indicate that graduate student mental health and well-being were substantially impacted during the COVID-19 pandemic. While approximately one-third of participants reported a formal mental health diagnosis, the majority exhibited symptoms of depression, anxiety, and stress. Nearly three-quarters reported depressive symptoms, and over half reported anxiety symptoms. Elevated stress was consistently observed on both the USS and DASS-S scales, and for about one-quarter of participants, mental health challenges were a persistent source of stress. Academic demands, procrastination, study-life balance, and work-related pressures were frequently cited as ongoing stressors. These findings align with earlier warnings from Evans et al. (1), who described graduate student mental health as a “growing crisis,” citing significantly elevated rates of depression and anxiety relative to the general population. More recent studies confirm these vulnerabilities, particularly as they were intensified during the COVID-19 pandemic (9, 14). Burnout and lack of work-life balance remain central stressors for graduate students (12, 57, 58). Social isolation and uncertainty —known pandemic stressors among the general population (59, 60) — appear to have exacerbated these effects in graduate students. The current data extend this work by documenting elevated stress levels in a Canadian graduate student sample. Notably, participants reported higher rates of depression and anxiety than those found in a nationally representative sample of Canadian young adults, where approximately 1 in 4 screened positive for depression, anxiety, and PTSD during the pandemic. Although PTSD was not assessed in our sample, the proportion of graduate students reporting depressive and anxiety symptoms was two to three times higher (56).

Our data are also in line with several previous studies demonstrating a greater impact of the pandemic on stress and mental health of female students. Female graduate students reported higher rates of anxiety, depression, general stress, and university stress compared to male graduate students. Women also reported higher levels of stress related to academic demands, mental health problems, and study-life balance compared to men. These results were expected as prior to the pandemic, female graduate students reported poorer mental health outcomes than males (1, 28). During the pandemic, young adult females reported higher levels of depression, anxiety, and stress compared to young adult males (54, 54). In one study conducted on graduate student mental health during the pandemic, female graduate students reported higher levels of anxiety compared to males (18). Thus, our findings support and extend these reports in demonstrating these differences in a Canadian graduate student sample.

These findings are extremely concerning as it shows that graduate students who are already prone to poor mental health outcomes faced significant threats to their mental health and well-being due to the pandemic. The appearance of these poor mental health outcomes can have detrimental effects to the physical health of graduate students (57); therefore, it is important to make mental health and well-being of graduate students a top priority at the institutional level.

### 4.2 Negative and positive outcomes of COVID-19 on graduate students

Graduate students reported numerous negative outcomes from the pandemic, with approximately one-third experiencing negative effects on their mental health, stress levels, social relationships, overall educational experience, and connectedness with family, friends, and loved ones. Beyond affecting mental health, the pandemic notably disrupted social connectivity in academic settings and the broader graduate school experience. Lockdown measures, implemented globally to mitigate COVID-19 transmission, led to widespread closures of university campuses, severely hindering peer interactions (24, 61). These necessary but restrictive measures caused delays in graduation, interruptions in research, cancellations of conference attendance, reduced motivation, disrupted communication with supervisors, inadequate workspaces, and poor time management (14, 18, 62). Additionally, the prevalent use of avoidance coping strategies may have further negatively influenced graduate students’ educational experiences, which aligns with this data identifying overall educational experience as one of the pandemic’s primary negative impacts.

Gender differences emerged in the negative impacts of COVID-19, with female graduate students disproportionately reporting greater negative impacts on social connectedness, career prospects, skills development, degree progression, motivation, engagement in course work, and research productivity. Young female students, in particular, have reported negative shifts in social networks due to the pandemic (63), alongside heightened vulnerability to loneliness (54, 64). Female graduate students have also reported greater challenges related to overall educational experience during the pandemic (62), consistent with the current findings. While graduate students broadly encountered pandemic-related difficulties, these findings highlight that female graduate students experienced these challenges more acutely. However, these results must be taken in the context of males being less likely to report mental health challenges (65).

These findings support the hypothesis that the COVID-19 pandemic negatively impacted multiple facets of graduate student life, especially mental health, social connectivity, and educational experiences. Given that social relationships are essential for good mental health, well-being, resilience, and stress mitigation (61), it is particularly concerning that many participants reported weakened social ties, potentially exacerbating mental health issues. Additionally, negative impacts on educational experiences are worrisome, as a prior research suggests these negative educational experiences contributed to graduate students’ decision against re-enrollment in the Fall term of 2020 (62). Nonetheless, while the pandemic introduced considerable challenges, it is also important to acknowledge that some individuals reported positive changes, such as improved work-life balance, increased flexibility in work and school schedules, and more time spent at home with family or children (66).

### 4.3 Coping with the COVID-19 pandemic

Individuals tend to engage with a variety of coping methods to manage stressors; however, the type of coping strategy employed can predict mental health outcomes. Emotion-focused coping methods such as avoidance, rumination, and blame have been associated with poorer mental health outcomes, whereas problem-solving coping methods promote more positive outcomes (67). However, these coping strategies may also function as socialization (e.g. social media use) and non-academic activities that serve as distractions from academic stress. Such distractions, although providing temporary relief, might indirectly negatively affect graduate students’ overall educational experience by reducing productivity or focus on academic responsibilities. Strong social relationships represent a positive coping strategy that can mitigate stressful experiences and support good mental health (61).

In our sample, graduate students reported watching TV, using social media, and working as their top three coping mechanisms, strategies that can be considered avoidant. Similar findings were reported among undergraduate students during the pandemic, who also relied on social media and other avoidant coping strategies such as sleeping and substance use (54). This is likely a COVID-specific finding, as social contacts were limited, and individuals were isolated in their homes for extended periods of time.

Binge-watching TV (i.e., watching multiple episodes of a TV show in a single viewing session) has been linked to increased depression and anxiety prior to the pandemic (68, 69). During the pandemic lockdown, individuals spent more time watching TV, and those experiencing anxiety symptoms were particularly likely to binge-watch as a coping mechanism (70). While a substantial portion of our sample watched TV, the study did not explicitly assess binge-watching behaviours, so it is difficult to identify if the coping strategy was indeed problematic or not. Thus, future studies should investigate if binge-watching TV is a problematic coping strategy for graduate students.

Social media was the second most common coping strategy. Frequent social media use has been linked to increased risks for depression, anxiety, and suicidal ideation (71). Recent evidence indicates frequent social media use during the pandemic correlated with elevated stress, depression, anxiety, and loneliness (72–74). Given that 25.7% of this sample heavily relied on social media for coping with the pandemic, future studies should explore its role as potentially detrimental or beneficial, especially since social media also serves as a vital form of socialization during periods of isolation.

Working was the third most common coping strategy reported by graduate students during the pandemic. While working as a coping mechanism may reflect avoidance and is associated with burnout in graduate students (58), it could also represent productive adaptation during the pandemic. Graduate students frequently balance heavy academic workloads alongside additional employment for financial reasons, increasing their susceptibility to burnout and poor work-life balance (12). Students who have a better work-life balance report a better quality of life, well-being, and are less likely to experience adverse mental health challenges than graduate students with poor work-life balance (1, 10). However, working could also be seen as a productive. During the pandemic, studies found that graduate students had more time to engage in other types of work such as the ability to attend conferences due to the virtual format of many conferences and increased time to work on writing manuscripts (14, 16). However, in this study, students were not explicitly asked what type of work they engaged in (e.g., graduate student work, part-time work, or both) and how many hours per week they were working on each task. Future studies should further clarify the nature of work-related coping to differentiate potentially avoidant or productive qualities.

Graduate students experience significant mental health challenges; however, it is important to identify the impacts of positive coping strategies, such as social support, which can buffer against the impact of stress on well-being (75). In this sample, 21.1% of graduate students used videoconferencing to connect with friends and family, reflecting active use of social support. Graduate students who use social support as a coping strategy report feeling like part of a supportive network where they are cared for and can seek help and support (12). A supportive network can consist of peers, family members, community members, mentors, and more. (12). In fact, there is a great amount of evidence to support that social support as a coping strategy does positively influence graduate student mental health (12, 29, 76, 77). Strong social supports can act as a buffer for high stress and psychological issues and works to cope with these issues (29, 76). Thus, fostering strong social support networks is likely crucial for enhancing graduate student well-being.

Gender differences influenced coping strategies during the pandemic, though these differences must be interpreted with caution and in context. In this study, women reported greater use of watching TV and using social media to manage pandemic-related stress. While these behaviours have been previously documented among females experiencing elevated stress (70), they may reflect differences in coping accessibility and socialization rather than inherent preferences. Social media likely served as a vital source of connection during periods of isolation, functioning as both a coping strategy and a means of maintaining social ties. However, frequent use of social media has also been linked to increased loneliness and adverse mental health outcomes (74), highlighting its complex role. Men in our study also reported use of avoidant coping strategies, including greater engagement in gaming. Similar trends have been found in undergraduate samples, with male students more likely to turn to video games during the pandemic (78). Importantly, these coping choices are influenced by gender norms, social expectations, and cultural patterns in help-seeking, and should not be viewed as fixed or universally representative. Future research should examine how coping patterns are shaped by intersecting factors (including gender identity, structural barriers, and social supports) to better understand the broader context of coping behaviours in graduate student populations.

### 4.4 Limitations and future directions

There are some limitations which must be acknowledged in this study. First, the data relied heavily on self- reporting which is subject to report bias, and it employed a cross-sectional design, limiting inferences of causality. In addition, the participant’s mood when they completed the survey may have impacted their responses. Women made up the majority of the sample, potentially skewing the proportion of reported mental health outcomes, pandemic-related impacts, and coping strategies. It is important to recognize that men typically attend college and graduate programs in lower numbers compared to women (30) and are often less likely to openly report mental health issues due to social and cultural factors (65). Consequently, the gender-specific findings must be interpreted with caution, as they may disproportionately reflect women’s experiences and underrepresent those of men. Future research investigating mental health among young adults should incorporate targeted recruitment strategies to increase male participation and carefully consider socio-cultural barriers that influence self-reporting behaviours.

Similarly, gender non-conforming graduate students were heavily underrepresented in the sample and due to insufficient sample size, had to be excluded from the statistical analyses. We acknowledge that gender non-conforming graduate students are an equity-deserving group and stress the importance of recruiting more gender non-conforming students to ensure a sample that is truly reflective of the graduate student population in Canada.

While our analyses focused on group comparisons and bivariate relationships, we acknowledge that multivariate regression models could offer additional precision by controlling for potential confounders such as age, year of study, or caregiving responsibilities. However, given the modest sample size and the number of individual coping variables examined, incorporating multiple covariates would have substantially increased model complexity and reduced statistical power. Future research with larger and more diverse samples should explore multivariate approaches to better understand the unique and combined contributions of demographic and contextual factors.

For future studies, it would also be of interest to look at different coping interventions (e.g., exercising indoors or connecting with friends and family members) on graduate students to explore if these interventions could help mitigate the poor mental health outcomes these students face, especially among female graduate students. It would also be interesting to look at different disciplines of graduate students and their mental health outcomes to identify if certain disciplines are more vulnerable to poor mental health outcomes than others. Future studies should also explore if mental health outcomes and coping strategies differ for graduate students of different disciplines (e.g., life science students vs business students). Lastly, a longitudinal design would help elucidate whether these outcomes influence longer-term mental health outcomes.

## 5 Conclusion

These findings confirm that the COVID-19 pandemic had a significant impact on the mental health and coping behaviours of Canadian graduate students. Compared to pre-pandemic data from Evans et al. (1), our results indicate even higher rates of depression, anxiety, and stress, reinforcing concerns that graduate student mental health is a growing crisis exacerbated by the pandemic. Notably, female graduate students reported worse mental health outcomes than their male peers and were more likely to engage in avoidant coping strategies such as social media use and passive distraction. Overall, avoidant coping was common across the sample, highlighting a critical need for targeted interventions that promote healthier, more adaptive strategies. These results underscore the urgency for Canadian universities to provide tailored mental health supports—particularly for women—and to implement institutional changes that prioritize student well-being. Promoting pro-social coping mechanisms and fostering a healthier work-life balance are essential steps in addressing the long-term mental health consequences of the pandemic and building resilience in graduate student populations.

## Data Availability

The raw data supporting the conclusions of this article will be made available by the authors, without undue reservation.

## References

[1] Evans TM Bira L Gastelum JB Weiss LT Vanderford NL. Evidence for a mental health crisis in graduate education. Nat Biotechnol. (2018) 36:282–4. doi: 10.1038/NBT.4089 29509732

[2] Garcia-Williams AG Moffitt L Kaslow NJ. Mental health and suicidal behavior among graduate students. Acad Psychiatry. (2014) 38:554–60. doi: 10.1007/S40596-014-0041-Y 24711096

[3] Leveque K Anseel F De Beuckelaer A van der Heyden J Gisle L. Work organization and mental health problems in PhD students. Res Policy. (2017) 46:868–79. doi: 10.1016/J.RESPOL.2017.02.008

[4] NHCA. Canadian Reference Group Executive Summary Spring 2019 (2019). Available online at: www.acha-ncha.org (Accessed April 28, 2022).

[5] Almeida DM Rush J Mogle J Piazza JR Cerino E Charles ST. Longitudinal change in daily stress across 20 years of adulthood: Results from the National Study of Daily Experiences. Dev Psychol. (2023) 59:515–23. doi: 10.1037/dev0001469 PMC9993073 36174182

[6] Trolle N Lund T Winding TN Labriola M. Perceived stress among 20–21 year-olds and their future labour market participation – an eight-year follow-up study. BMC Public Health. (2017) 17:287. doi: 10.1186/s12889-017-4179-x 28359276 PMC5374618

[7] Liu Y Frazier PA Porta CM Lust K. Mental health of US undergraduate and graduate students before and during the COVID-19 pandemic: Differences across sociodemographic groups. Psychiatry Res. (2022) 309:114428. doi: 10.1016/j.psychres.2022.114428 35131558 PMC8805912

[8] Wang X Hegde S Son C Keller B Smith A Sasangohar F. Investigating mental health of US college students during the COVID-19 pandemic: cross-sectional survey study. J Med Internet Res. (2020) 22:e22817. doi: 10.2196/22817 32897868 PMC7505693

[9] Browning MHEM Larson LR Sharaievska I Rigolon A McAnirlin O Mullenbach L. Psychological impacts from COVID-19 among university students: Risk factors across seven states in the United States. PloS One. (2021) 16:e0245327. doi: 10.1371/journal.pone.0245327 33411812 PMC7790395

[10] Yusuf S Joseph P Rangarajan S Islam S Mente A Hystad P. Modifiable risk factors, cardiovascular disease, and mortality in 155 722 individuals from 21 high-income, middle-income, and low-income countries (PURE): a prospective cohort study. Lancet (London England). (2020) 395:795–808. doi: 10.1016/S0140-6736(19)32008-2 31492503 PMC8006904

[11] Linden B Stuart H Ecclestone A. Trends in post-secondary student stress: A pan-Canadian study. Can J Psychiatry Rev Can Psychiatr. (2023) 68:521–30. doi: 10.1177/07067437221111365 PMC10408557 35791667

[12] Hosek AM Waldbuesser C. Graduate student identity salience and mental health. Ohio Commun J. (2020) 58:132–44.

[13] Lee J. Mental health effects of school closures during COVID-19. Lancet Child Adolesc Health. (2020) 4:421. doi: 10.1016/S2352-4642(20)30109-7 32302537 PMC7156240

[14] Varadarajan J Brown AM Chalkley R. Biomedical graduate student experiences during the COVID-19 university closure. PloS One. (2021) 16:e0256687. doi: 10.1371/JOURNAL.PONE.0256687 34529681 PMC8445460

[15] Cai Q LeBouef S Savage M Dworkin J. What happened when COVID-19 shut down in-person higher education? Parents speak out. About Campus: Enrich Stud Learn Exp. (2022) 26:26–34. doi: 10.1177/10864822221082695

[16] Bradham JL Umaña MN. Perceptions by early career tropical researchers on the impact of COVID-19 six months into the pandemic. Biotropica. (2021) 53:1250–4. doi: 10.1111/BTP.13004 PMC8444713 34548674

[17] Suart C Nowlan Suart T Graham K Truant R. When the labs closed: Graduate students’ and postdoctoral fellows’ experiences of disrupted research during the COVID-19 pandemic. FACETS. (2021) 6:966–97. doi: 10.1139/facets-2020-0077

[18] Chirikov I Soria KM Horgos B Jones-White D. UC Berkeley SERU Consortium Reports Title Undergraduate and Graduate Students’ Mental Health During the COVID-19 Pandemic (2020). Available online at: https://escholarship.org/uc/item/80k5d5hw (Accessed April 21, 2025).

[19] Dial LA DeNardo FA Fevrier B Morgan AL Du C Tucker RM. Comparing mental health and well-being of US undergraduate and graduate students during the early stages of the COVID-19 pandemic. J Am Coll Health: J ACH. (2023) 71:2775–85. doi: 10.1080/07448481.2021.1996372 34788587

[20] Zhen F Graves-Boswell T Rugh MS Clough JM. I have no idea how I will get a stipend”: the impact of COVID-19 on graduate students’ financial security. Front Educ. (2024) 9:1235291/full. doi: 10.3389/feduc.2024.1235291/full

[21] Tozini K Castiello-Gutierrez S. COVID-19 and international students: examining perceptions of social support, financial well-being, psychological stress, and university response. J Coll Stud Dev. (2022) 63:134–50. doi: 10.1353/csd.2022.0011

[22] Jairam D Kahl DH. Navigating the doctoral experience: The role of social support in successful degree completion. Int J Doctor Stud. (2012) 7:311–29. doi: 10.28945/1700

[23] Best M Williams J. 2013 student Health Survey Report National College Health Assessment (NCHA). Queen’s University (2013).

[24] Korbel JO Stegle O. Effects of the COVID-19 pandemic on life scientists. Genome Biol. (2020) 21:1–5. doi: 10.1186/S13059-020-02031-1/FIGURES/1 PMC7212246 32393316

[25] Gilmore J Wofford AM Maher MA. The flip side of the attrition coin: Faculty perceptions of factors supporting graduate student success. Int J Doctor Stud. (2016) 11:419–39. http://www.informingscience.org/Publications/3618.

[26] King MF. Ph.D. completion and attrition: Analysis of baseline demographic data from the Ph.D. Completion Project. Council Grad Schools. (2008).

[27] Sowell R Allum J Okahana H. Doctoral Initiative on Minority Attrition and Completion. Washington DC: Council of Graduate Schools (2015).

[28] Lipson SK Raifman J Abelson S Reisner SL. Gender minority mental health in the U.S.: results of a national survey on college campuses. Am J Prev Med. (2019) 57:293–301. doi: 10.1016/J.AMEPRE.2019.04.025 31427032

[29] Wang P Xiong Z Yang H. Relationship of mental health, social support, and coping styles among graduate students: evidence from Chinese universities. Iran J Public Health. (2018) 47:689. 29922611 PMC6005982

[30] Statistics Canada. Proportion of male and female postsecondary graduates, by field of study and International Standard Classification of Education. (2021) Available online at: https://www150.statcan.gc.ca/t1/tbl1/en/tv.action?pid=3710013502.

[31] Boivin N Täuber S Mahmoudi M. Overcoming gender bias in STEM. Trends Immunol. (2024) 45:483–5. doi: 10.1016/j.it.2024.05.004 38862366

[32] Llorens A Tzovara A Bellier L Bhaya-Grossman I Bidet-Caulet A Chang WK. Gender bias in academia: A lifetime problem that needs solutions. Neuron. (2021) 109:2047–74. doi: 10.1016/j.neuron.2021.06.002 PMC8553227 34237278

[33] Parmaxi A Christou E Fernández Valdés J Puente Hevia DM Perifanou M Economides AA. Gender equality in science, technology, engineering and mathematics: Industrial vis-a-vis academic perspective. Discov Educ. (2024) 3:3. doi: 10.1007/s44217-023-00082-7

[34] Oliveira DFM Ma Y Woodruff TK Uzzi B. Comparison of national institutes of health grant amounts to first-time male and female principal investigators. JAMA. (2019) 321:898–900. doi: 10.1001/jama.2018.21944 30835300 PMC6439593

[35] Mason S Bond M Ledger S. How motherhood enhances and strains doctoral research/ers. J Further High Educ. (2023) 47:1087–105. doi: 10.1080/0309877X.2023.2218274

[36] Llorens A Tzovara A Bellier L Bhaya-Grossman I Bidet-Caulet A Chang WK. Gender bias in academia: A lifetime problem that needs solutions. Neuron. (2021) 109(13):2047–74. doi: 10.1080/07448481.2018.1434780 PMC8553227 34237278

[37] Lazarus RS Folkman S. Stress, Appraisal, and Coping. Princeton, New Jersey: Springer Publishing Company (1984).

[38] Dyson R Renk K. Freshmen adaptation to university life: Depressive symptoms, stress, and coping. J Clin Psychol. (2006) 62:1231–44. doi: 10.1002/jclp.20295 16810671

[39] Pascoe MC Hetrick SE Parker AG. The impact of stress on students in secondary school and higher education. Int J Adol Youth. (2020) 25:104–12. doi: 10.1080/02673843.2019.1596823

[40] Davies EB Wardlaw J Morriss R Glazebrook C. An experimental study exploring the impact of vignette gender on the quality of university students’ mental health first aid for peers with symptoms of depression. BMC Public Health. (2016) 16:1–11. doi: 10.1186/S12889-016-2887-2/FIGURES/2 26911725 PMC4766614

[41] Pattyn E Verhaeghe M Bracke P. The gender gap in mental health service use. Soc Psychiatry Psychiatr Epidemiol. (2015) 50:1089–95. doi: 10.1007/s00127-015-1038-x 25788391

[42] Zochil ML Thorsteinsson EB. Exploring poor sleep, mental health, and help-seeking intention in university students (2020). doi: 10.1111/AJPY.12160.

[43] Ickes MJ Brown J Reeves B Zephyr PMD. Differences between undergraduate and graduate students in stress and coping strategies. Californian J Health Promot. (2015) 13:13–25. doi: 10.32398/CJHP.V13I1.1810

[44] Lovibond PF Lovibond SH. The structure of negative emotional states: Comparison of the Depression Anxiety Stress Scales (DASS) with the Beck Depression and Anxiety Inventories. Behav Res Ther. (1995) 33:335–43. doi: 10.1016/0005-7967(94)00075-U 7726811

[45] Laranjeira C Querido A Sousa P Dixe MA. Assessment and Psychometric Properties of the 21-Item Depression Anxiety Stress Scale (DASS-21) among Portuguese Higher Education Students during the COVID-19 Pandemic. Eur J Invest health Psychol Educ. (2023) 13:2546–60. doi: 10.3390/ejihpe13110177 PMC10670895 37998067

[46] Beck AT Epstein N Brown G Steer RA. An inventory for measuring clinical anxiety: psychometric properties. J consult Clin Psychol. (1988) 56:893. doi: 10.1037/0022-006X.56.6.893 3204199

[47] Leyfer OT Ruberg JL Woodruff-Borden J. Examination of the utility of the Beck Anxiety Inventory and its factors as a screener for anxiety disorders. J Anxiety Disord. (2006) 20:444–58. doi: 10.1016/j.janxdis.2005.05.004 16005177

[48] Oh H Park K Yoon S Kim Y Lee SH Choi YY. Clinical utility of beck anxiety inventory in clinical and nonclinical Korean samples. Front Psychiatry. (2018) 9:666. doi: 10.3389/fpsyt.2018.00666 30564158 PMC6288426

[49] Beck AT Ward CH Mendelson M Mock J Erbaugh J. An inventory of measuring depression. Arch Gen Psychiatry. (1961) 4:561–71. doi: 10.1001/archpsyc.1961.01710120031004 13688369

[50] Eser MT Aksu G. Beck depression inventory-II: A study for meta analytical reliability generalization. Pegem J Educ Instruct. (2021) 11.

[51] Kokou-Kpolou CK Jumageldinov A Park S Nieuviarts N Noorishad P-G Cénat JM. Prevalence of depressive symptoms and associated psychosocial risk factors among French university students: the moderating and mediating effects of resilience. Psychiatr Q. (2021) 92:443–57. doi: 10.1007/s11126-020-09812-8 32804341

[52] Stallman HM Hurst CP. The university stress scale: measuring domains and extent of stress in university students. Aust Psychol. (2016) 51:128–34. doi: 10.1111/AP.12127

[53] Stevens C Schneider E Bederman-Miller P Arcangelo K. Exploring the Relationship between Emotional Intelligence and Academic Stress among Students at a Small, Private College. Contemp Issues Educ Res. (2019) 12:93–102. doi: 10.19030/cier.v12i4.10322

[54] Prowse R Sherratt F Abizaid A Gabrys RL Hellemans KGC Patterson ZR. Coping with the COVID-19 pandemic: examining gender differences in stress and mental health among university students. Front Psychiatry. (2021) 12:650759/BIBTEX. doi: 10.3389/FPSYT.2021.650759/BIBTEX 33897499 PMC8058407

[55] Faul F Erdfelder E Lang A-G Buchner A. G*Power 3: A flexible statistical power analysis program for the social, behavioral, and biomedical sciences. Behav Res Methods. (2007) 39:175–91. doi: 10.3758/BF03193146 17695343

[56] Statistics Canada. The Daily — Survey on COVID-19 and Mental Health, February to May 2021 (2021). Available online at: https://www150.statcan.gc.ca/n1/daily-quotidien/210927/dq210927a-eng.htm (Accessed April 15, 2025).

[57] Gallea JI Medrano LA Morera LP. Work-related mental health issues in graduate student population. Front Neurosci. (2021) 15:593562. doi: 10.3389/FNINS.2021.593562 33867910 PMC8049290

[58] Pascale AB. Co-Existing Lives. In: Understanding and Facilitating Graduate Student Sense of Belonging. Journal of Student Affairs Research and Practice (2018). doi: 10.1080/19496591.2018.1474758

[59] Pfefferbaum B North CS. Mental health and the covid-19 pandemic. New Engl J Med. (2020) 383:510–2. doi: 10.1056/NEJMP2008017/SUPPL_FILE/NEJMP2008017_DISCLOSURES.PDF 32283003

[60] Rossi R Socci V Talevi D Mensi S Niolu C Pacitti F. COVID-19 pandemic and lockdown measures impact on mental health among the general population in Italy. Front Psychiatry. (2020) 11:790/BIBTEX. doi: 10.3389/FPSYT.2020.00790/BIBTEX 32848952 PMC7426501

[61] Filho WL Wall T Rayman-Bacchus L Mifsud M Pritchard DJ Lovren VO. Impacts of COVID-19 and social isolation on academic staff and students at universities: a cross-sectional study. BMC Public Health. (2021) 21:1–19. doi: 10.1186/S12889-021-11040-Z/TABLES/6 34167494 PMC8223197

[62] Soria KM. UC Berkeley SERU Consortium Reports Title Graduate and Professional Students’ Financial Hardships During the COVID-19 Pandemic: Evidence from the gradSERU COVID-19 Survey (2020). Available online at: https://escholarship.org/uc/item/8wv3d1cc (Accessed October 27, 2021).

[63] Elmer T Mepham K Stadtfeld C. Students under lockdown: Comparisons of students’ social networks and mental health before and during the COVID-19 crisis in Switzerland. PloS One. (2020) 15. doi: 10.1371/JOURNAL.PONE.0236337 PMC7377438 32702065

[64] Liu H Zhang M Yang Q Yu B. Gender differences in the influence of social isolation and loneliness on depressive symptoms in college students: a longitudinal study. Soc Psychiatry Psychiatr Epidemiol. (2020) 55:251–7. doi: 10.1007/S00127-019-01726-6 31115597

[65] Chatmon BN. Males and mental health stigma. Am J Men’s Health. (2020) 14:1557988320949322. doi: 10.1177/1557988320949322 32812501 PMC7444121

[66] Ipsen C van Veldhoven M Kirchner K Hansen JP. Six key advantages and disadvantages of working from home in Europe during COVID-19. Int J Environ Res Public Health. (2021) 18. doi: 10.3390/ijerph18041826 PMC7917590 33668505

[67] Matheson K Anisman H. Systems of coping associated with dysphoria, anxiety and depressive illness: a multivariate profile perspective. Stress (Amsterdam Netherlands). (2003) 6:223–34. doi: 10.1080/10253890310001594487 13129815

[68] Ahmed A A-A M. New Era of TV-Watching Behavior: Binge Watching and its Psychological Effects - Media Watch Journal. Media Watch. (2017) 8:192–207. https://www.mediawatchjournal.in/new-era-of-tv-watching-behavior-binge-watching-and-its-psychological-effects-2/ (Accessed April 16, 2022).

[69] Tukachinsky R Eyal K. The psychology of marathon television viewing: antecedents and viewer involvement. Mass Commun Soc. (2018) 21:275–95. doi: 10.1080/15205436.2017.1422765

[70] Boursier V Musetti A Gioia F Flayelle M Billieux J Schimmenti A. Is watching TV series an adaptive coping strategy during the COVID-19 pandemic? Insights from an Italian community sample. Front Psychiatry. (2021) 12:599859/BIBTEX. doi: 10.3389/FPSYT.2021.599859/BIBTEX 33967845 PMC8097049

[71] Karim F Oyewande A Abdalla LF Chaudhry Ehsanullah R Khan S. Social media use and its connection to mental health: A systematic review. Cureus. (2020) 12. doi: 10.7759/CUREUS.8627 PMC7364393 32685296

[72] Gao J Zheng P Jia Y Chen H Mao Y Chen S. Mental health problems and social media exposure during COVID-19 outbreak. PloS One. (2020) 15. doi: 10.1371/JOURNAL.PONE.0231924 PMC7162477 32298385

[73] Zhao N Zhou G. Social media use and mental health during the COVID-19 pandemic: moderator role of disaster stressor and mediator role of negative affect. Appl Psychol Health Well-Being. (2020) 12:1019–38. doi: 10.1111/APHW.12226 PMC7536964 32945123

[74] Ghanayem LK Shannon H Khodr L McQuaid RJ Hellemans KGC. Lonely and scrolling during the COVID-19 pandemic: understanding the problematic social media use and mental health link among university students. Front Psychiatry. (2024) 15:1247807. doi: 10.3389/fpsyt.2024.1247807 38356913 PMC10864490

[75] McQuaid RJ McInnis OA Paric A Al-Yawer F Matheson K Anisman H. Relations between plasma oxytocin and cortisol: The stress buffering role of social support. Neurobiol Stress. (2016) 3:52–60. doi: 10.1016/j.ynstr.2016.01.001 27981177 PMC5146198

[76] McKinney B. Associations among Social Support, Life Purpose and Graduate Student Stress. VAHPERD J. (2017) 38:4–10. https://go-gale-com.proxy.library.carleton.ca/ps/i.do?p=AONE&sw=w&issn=07394586&v=2.1&it=r&id=GALE%7CA542801438&sid=googleScholar&linkaccess=fulltext (Accessed October 27, 2021).

[77] Allen SF Stevenson J Lazuras L Akram U. The role of the COVID-19 pandemic in altered psychological well-being, mental health and sleep: an online cross-sectional study. Psychol Health Med. (2022) 27(2):343–51. doi: 10.1080/13548506.2021.1916963 33878999

[78] Almomani EY Qablan AM Almomany AM Atrooz FY. The coping strategies followed by university students to mitigate the COVID-19 quarantine psychological impact. Curr Psychol (New Brunswick N.J.). (2021) 40:5772–81. doi: 10.1007/s12144-021-01833-1 PMC8106545 33994758

